# Supplementary figures and images for: Kinesin-1, -2, and -3 motors use family-specific mechanochemical strategies to effectively compete with dynein during bidirectional transport
Source: eLife. 2022 Sep 20;11:e82228. doi: 10.7554/eLife.82228 (PMC9545524; doi:10.7554/eLife.82228)

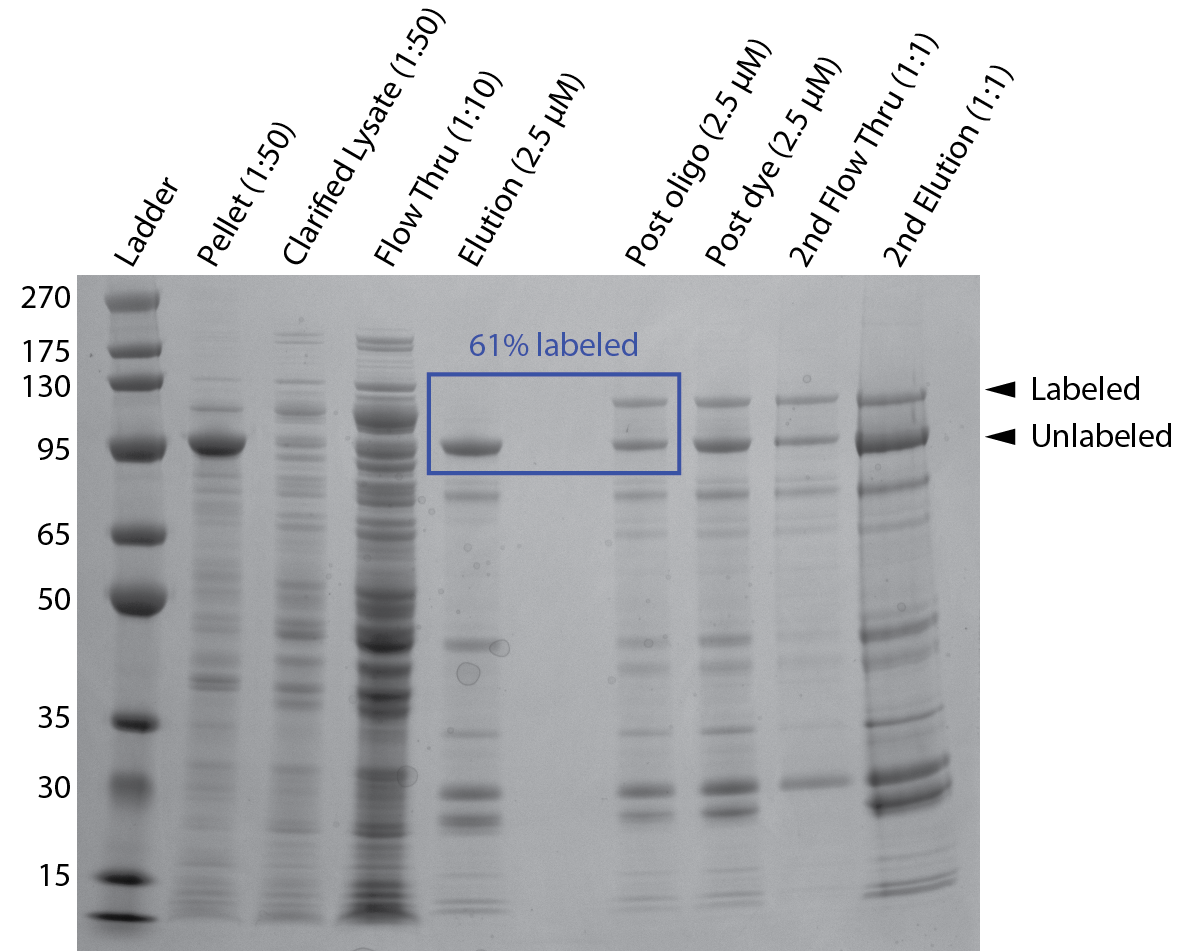

Supplement: Figure 1—figure supplement 1—source data 1. [file elife-82228-fig1-figsupp1-data1.zip › Gels/K560SNAP_Purification_Gel.png]

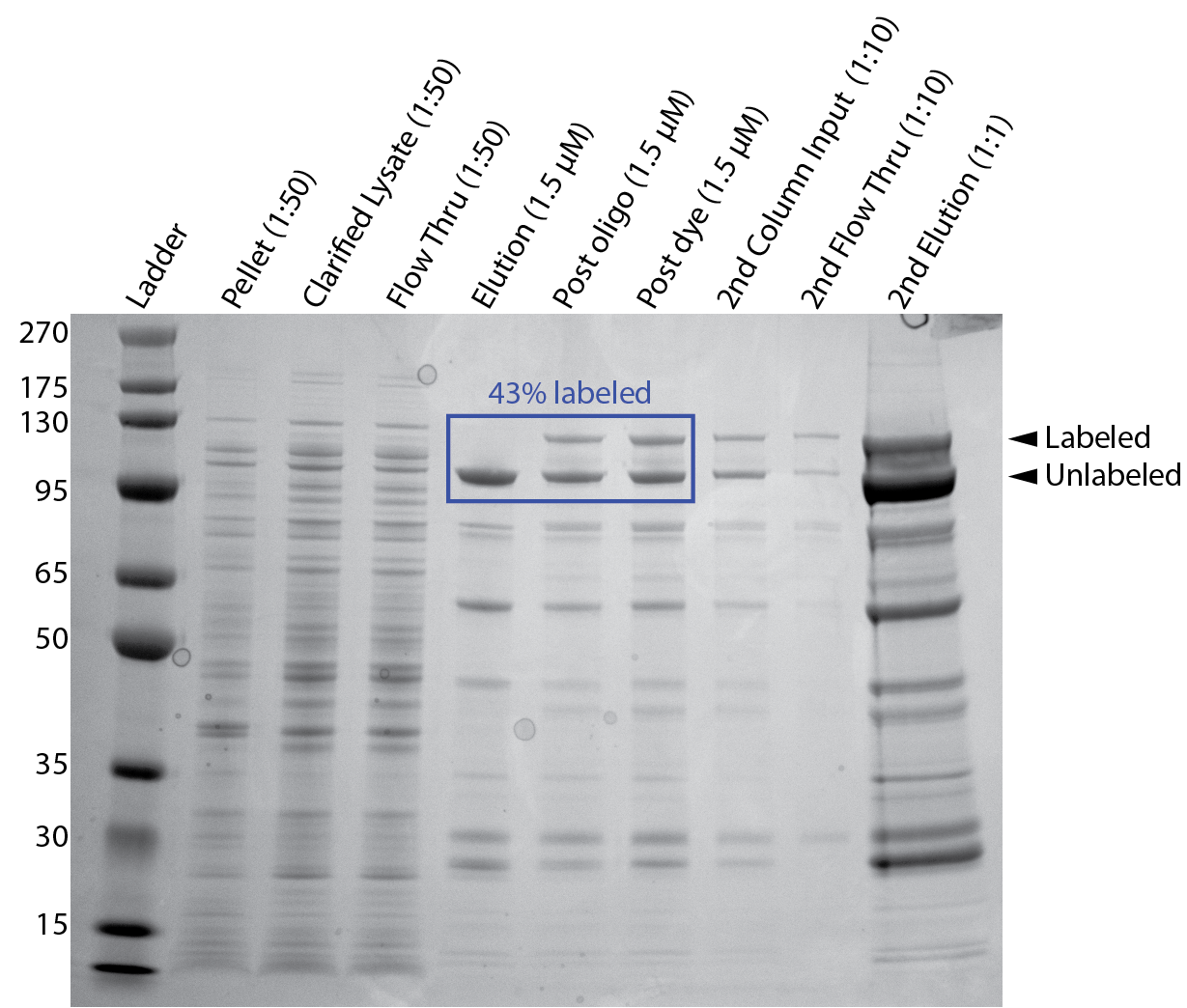

Supplement: Figure 1—figure supplement 1—source data 1. [file elife-82228-fig1-figsupp1-data1.zip › Gels/3A560SNAP_Purification_Gel.png]

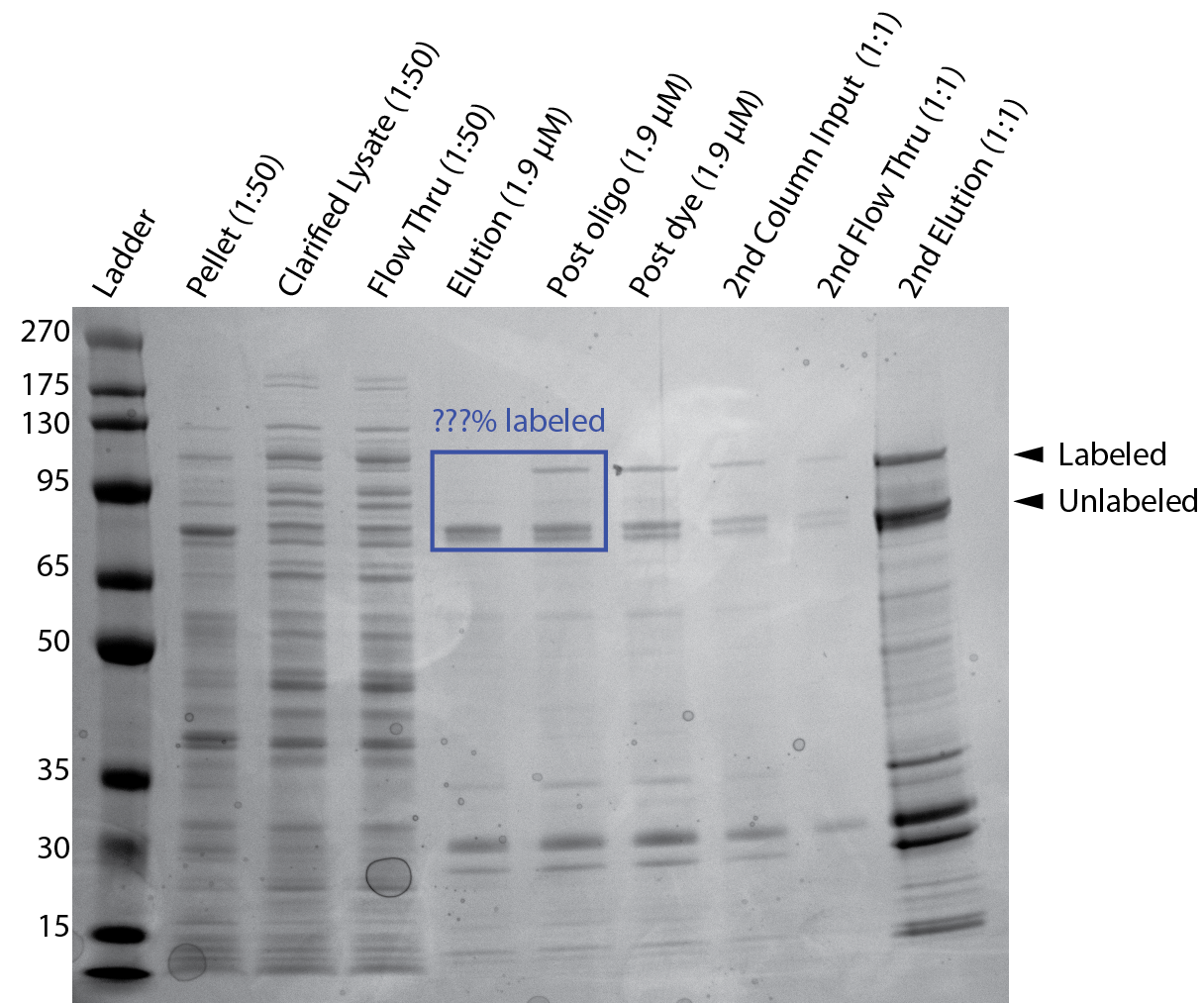

Supplement: Figure 1—figure supplement 1—source data 1. [file elife-82228-fig1-figsupp1-data1.zip › Gels/AviKif1aSNAP_Purification_Gel.png]
